# Tuning a 96-Well Microtiter Plate Fluorescence-Based Assay to Identify AGE Inhibitors in Crude Plant Extracts

**DOI:** 10.3390/molecules181114320

**Published:** 2013-11-19

**Authors:** Luc Séro, Lionel Sanguinet, Patricia Blanchard, Bach Tai Dang, Sylvie Morel, Pascal Richomme, Denis Séraphin, Séverine Derbré

**Affiliations:** 1 EA 921 SONAS, Université d'Angers, SFR QUASAV 4207, Angers 49045, France; 2 MOLTECH Anjou, UMR 6200 CNRS, UFR Sciences, Université d'Angers, Angers 49045, France

**Keywords:** advanced glycation end-products, automation, fluorescence, natural products, pentosidine, plant extract screening, vesperlysines

## Abstract

Advanced glycation end-products (AGEs) are involved in the pathogenesis of numerous diseases. Among them, cellular accumulation of AGEs contributes to vascular complications in diabetes. Besides using drugs to lower blood sugar, a balanced diet and the intake of herbal products potentially limiting AGE formation could be considered beneficial for patients’ health. The current paper presents a simple and cheap high-throughput screening (HTS) assay based on AGE fluorescence and suitable for plant extract screening. We have already implemented an HTS assay based on vesperlysines-like fluorescing AGEs quickly (24 h) formed from BSA and ribose under physiological conditions. However, interference was noted when fluorescent compounds and/or complex mixtures were tested. To overcome these problems and apply this HTS assay to plant extracts, we developed a technique for systematic quantification of both vesperlysines (λ_exc_ 370 nm; λ_em_ 440 nm) and pentosidine-like (λ_exc_ 335 nm; λ_em_ 385 nm) AGEs. In a batch of medicinal and food plant extracts, hits were selected as soon as fluorescence decreased under a fixed threshold for at least one wavelength. Hits revealed during this study appeared to contain well-known and powerful anti-AGE substances, thus demonstrating the suitability of this assay for screening crude extracts (0.1 mg/mL). Finally, quercetin was found to be a more powerful reference compound than aminoguanidine in such assay.

## 1. Introduction

Advanced glycation end-products (AGEs) are formed during non-enzymatic reactions involving proteins and sugars, *i.e.* the Maillard or browning reaction [1]. Briefly, the Maillard reaction can be divided into two stages. The early glycation is reversible and involves the production of a Schiff base from the carbonyl group of a reducing sugar and the primary amino groups of a protein (lysine, arginine). The imine adduct undergoes rearrangement to form Amadori products such as HbA_1c_ (glycated haemoglobin), which is widely used as a diabetes control marker [2]. During the late stage, complex irreversible oxidation, condensation and cyclisation reactions lead to AGEs via intra- and intermolecular protein crosslinkage [3,4].

When the organism can no longer synthesise or properly use insulin, chronic hyperglycaemia occurs, which is responsible for complications in diabetes through AGE formation. [5]. These complications [6,7] include atherosclerosis [8], nephropathies [9] and cataracts [10]. According to the World Health Organization (WHO), 346 million people suffer from diabetes worldwide, whereas deaths due to this disease will double between 2005 and 2030. To avoid such complications, insulin or oral antidiabetic drugs are used to lower blood sugar levels [11] and thus AGE formation [6]. However, antioxidants acting by radical scavenging or metal chelation [12,13,14] and compounds able to trap dicarbonyl species or break AGEs may also partially contribute to limiting the amount of AGEs and related complications [3]. From a broader perspective, such substances could also be useful for limiting age-related pathologies. Indeed, AGEs are also involved in neurological diseases like the Alzheimer's disease [15] and joint diseases [16]. More generally, they cause aging of many tissues as they accumulate in organisms over time [17]. These considerations have prompted the search for AGE inhibitors. Pure products may thus be considered as potential drugs. But when complex standardized mixtures are extracted from medicinal or food plants, these substances could be considered as dietary supplements [3,14,18,19]. Indeed, over the last decade, rediscovery of the connection between plants and their environment through phytochemicals has given rise to a new generation of botanical therapeutics *sensu lato* in the form of multi-component herbal preparations or dietary supplements [20,21]. Concerning diabetes, a wide variety of herbs is used worldwide to prevent this disease and its consequences. Some of them could contribute to reducing the formation of AGEs such as natural guanidines (*i.e.*, galegine isolated from *Galega officinalis* L.) [22,23] or antioxidant polyphenols [13,14]. In this setting, suitable herbal dietary supplements, combined with antidiabetic drugs, a balanced diet or herbal teas, could also help to prevent AGE formation.

**Figure 1:**
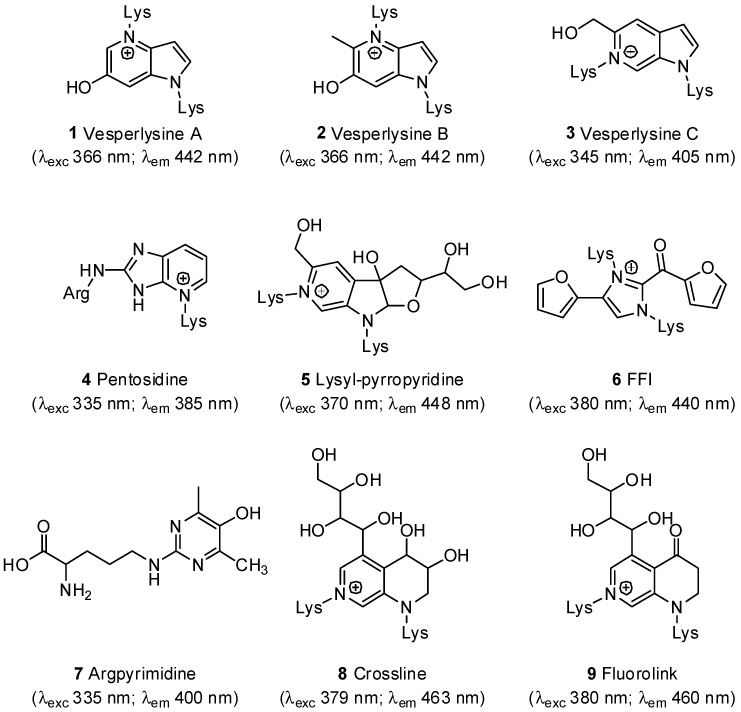
Structures of the main fluorescent AGEs **1**–**9** and their fluorescent properties in neutral aqueous media [31,32,33,34,35,36].

Addressing this issue, plant extracts have been widely evaluated for their ability to prevent AGE formation *in vitro* [14]. A simple chemical assay based on AGE fluorescence (Figure 1) obtained from BSA and a previously described mixture of glucose/fructose [24] or its successive modifications have often been used [25]. However, many difficulties were encountered when this screening system was applied to natural extracts containing fluorescent products. AGE formation is assessed by arithmetic subtraction of the extract’s fluorescence from those of the extract/AGEs mixture. Aberrant negative values may, however, be obtained [26]. Fluorescent (Figure 1) and non-fluorescent AGEs could be distinguished [25]. Other methods have been proposed to detect AGE inhibitors without using fluorescence detection. Immunochemical assays (e.g., ELISA) [27,28,29] require expensive antibodies and are unsuitable for a rapid screening, while the procedure proposed by Fatima *et al.*, based on the ability of AGE inhibitors to protect RNase against sugar-induced inactivation of the enzyme [30], has the same drawbacks.

Here we propose to improve screening using an anti-AGE fluorescence-based assay previously described for screening pure products [37] and to tailor it to plant extracts. We thus examined the fluorescence spectrum of AGEs formed from bovine serum albumin (BSA) and ribose, *i.e.*, the protein and sugar used in this assay, allowing an anti-AGE screening after only 24 h, as previously described [37]. Fluorescence at λ_exc_ 370 nm and λ_em_ 440 nm is widely used as an *in vitro* [26,37] or *in vivo* [38] marker of Maillard product levels in proteins. In fact, AGE fluorescence depends on the proteins and sugars/dicarbonylated compounds involved in heterocycle formation via intra- and inter-molecular crosslinking [14]. As secondary metabolites from plant extracts often have UV maxima between 300 and 400 nm, interference with intrinsic AGE fluorescence cannot be excluded. We thus postulated that more reliable results could be obtained through two measurements of AGE fluorescence (λ_exc_ 370 nm; λ_em_ 440 nm for vesperlysines-like AGEs and λ_exc_ 335 nm; λ_em_ 385 nm for pentosidine-like AGEs) [39] formed under previously described conditions [37] and data comparison.

## 2. Results and Discussion

### 2.1. Fluorescence Properties of AGEs Produced from BSA and Ribose

**Figure 2:**
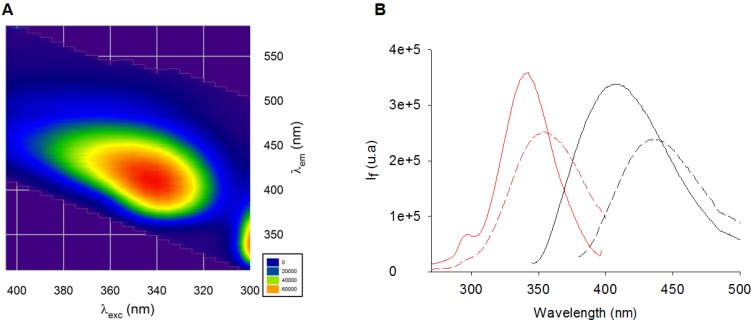
(**A**) Total fluorescence of AGEs formed from BSA (10 mg/mL) and ribose (0.5 M). The 2D contour plot shows variations in the emission spectrum as a function of the excitation wavelength. The fluorescence intensity increased from blue to red. This graph indicates that both pentosidine-like (λ_exc_ 335 nm; λ_em_ 385 nm) and vesperlysines-like (λ_exc_ 370 nm; λ_em_ 440 nm) AGEs were the main fluorescing AGEs obtained after incubation; (**B**) Excitation (red) and emission (black) fluorescence spectra of vesperlysines-like (dotted, λ_exc_ 370 nm; λ_em_ 440 nm) and pentosidine-like (bold, λ_exc_ 335 nm; λ_em_ 400 nm) AGEs formed from BSA and ribose.

A literature survey showed that AGEs were detected in former fluorescence-based assays using various excitation/emission wavelengths. First, in order to evaluate AGE formation *in vitro* when potential inhibitors were added to a given solution, Vinson and Howard used excitation and emission wavelengths of 350 and 450 nm, but without any explanation to justify this choice [24]. However, as depicted in Figure 1, those wavelengths are not specific to well-characterized fluorescent AGEs. Without any more explanation, similar assays using AGE detection at 415–460 nm (λ_exc_ 350–385 nm) [40] or 385 nm (λ_exc_ 335 nm) were conducted to quantify vesperlysines-like AGEs [26,41,42,43,44] or pentosidine-like AGEs, respectively [45]. The structure and fluorescence properties of AGEs both depend on amino acids (*i.e.*, lysine and/or arginine) and sugars/carbonyl compounds (*i.e.*, glucose, fructose, ribose and/or glyoxal, methylglyoxal, 3-deoxyglucosone), which are involved in their formation [14]. We thus studied the fluorescence properties of AGEs formed from BSA (10 mg/mL) and ribose (0.5 M) for 24 h at 37 °C in a physiological environment. In these conditions, variations in the emission spectrum as a function of the excitation wavelength were measured and a 2D contour plot was drawn up (Figure 2A). The graph showed high fluorescence around 440 nm (λ_exc_ 370 nm), but the highest signal was found between 390 and 440 nm (λ_exc_ 330 to 350 nm) and could correspond to the excitation and emission wavelengths of pentosidine **4** (λ_exc_ 335 nm; λ_em_ 385 nm). More precisely, as shown in Figure 2B, maximum emission occurred at 440 nm when 370 nm was used as excitation wavelength. These fluorescence properties were in agreement with those of most well-known fluorescent AGEs, especially vesperlysines-like ones **1** and **2**. Similarly, when 335 nm was chosen as excitation wavelength, maximum fluorescence was detected around 400 nm on the emission spectrum, suggesting the formation of pentosidine **4** or argpyrimidine **7** -like AGEs (Figure 2B). Previously, when AGEs were quantified at 440 nm (λ_exc_ 370 nm), we noticed many interferences when the tested products were phenolic acids or coumarins [37]. In such cases, we assumed that measurement of pentosidine-like AGEs could prevent such interference. For screening, we thus suggest that both vesperlysines-like (λ_exc_ 370 nm; λ_em_ 440 nm) and pentosidine-like (λ_exc_ 335 nm; λ_em_ 385 nm) AGEs should be systematically quantified and selecting hits as soon as AGEs percentage would decrease under a fixed-threshold at least for one type of AGEs. A statistical analysis was required for both measurements so that HTS could be carried out.

### 2.2. Statistical Analysis of the Automated High Throughput Screening Assay Results

The objective here was to improve an existing automated high-throughput screening (HTS) assay and tailor it for crude extract screening. As for vesperlysines-like AGE quantification (λ_exc_ 370 nm; λ_em_ 440 nm) [37], a statistical analysis was required to check whether the difference between the positive signal (*i.e.*, AGE formation from BSA and ribose) and negative signal (*i.e.*, BSA alone) could allow a single measurement without replicates when pentosidine-like AGEs (λ_exc_ 335 nm; λ_em_ 385 nm) were evaluated. The assay quality as well as the signal homogeneity were first evaluated through a Z′-factor calculation [46]. This popularly used Z-factor-based QC criterion is most suitable for the strong or very strong positive controls. That is why the strictly standardized mean difference (SSMD)-based criterion, recently proposed by Zhang *et al.* was also calculated. An assay with SSMD ≥ 3 (SSMD ≤ −3) passes QC and fails otherwise [47,48].

As depicted in Table 1, after 24 h incubation at 37 °C in physiological conditions, the measurements of vesperlysines-like or pentosidine-like AGEs formed from BSA (10 mg/mL) and ribose (0.5 M) indicated similar Z'-factors of 0.70 and 0.75, respectively. Those values (>0.5) could be regarded as indicators of very good screening assays, allowing measurements after a single experiment. This was confirmed by SSMD values, 9.9 ± 1.5 and 11.1 ± 2.2 respectively when vesperlysines-like and pentosidine-like AGEs were quantified. According to Zhang [47], values > 7 indicate an extremely strong control. Dealing with repeatability, the plate-to-plate variability was low and similar when vesperlysines- or pentosidine-like AGEs were taken into account. In terms of reproducibility, the day-to-day variability also appeared to be analogous and satisfactory.

### 2.3. Screening Anti-AGE Activity of a Small Library of Natural Products and Validation of Hits by Dose-Effect Curves Using Two Fluorescence Measurements

Only vesperlysines-like AGEs were quantified (λ_exc_ 370 nm; λ_em_ 440 nm) in the automated anti-AGE HTS assay we previously developed [37]. However, using this single wavelength detection, unexpected results were obtained for some NPs. Dose-effect curves were plotted to gain insight into this phenomenon. As expected, we noted that sigmoidal curves could not be obtained for phenolic acids (caffeic and chlorogenic acids), fluorescent coumarins or emetin alkaloid. These results clearly highlighted that fluorescence interference occurred between AGEs and the tested products.

**Table 1:** Statistical analysis and comparison of both anti-vesperlysines-like and anti-pentosidine-like AGE assays. BSA (10 mg/mL) was incubated with d-ribose (0.5 M) in phosphate buffer to obtain positive controls. BSA alone was used as blank control. Solutions were incubated in 96-well microtiter plates at 37 °C for 24 h before vesperlysines-like (λ_exc_ 370 nm, λ_em_ 440 nm) and pentosidine-like (λ_exc_ 335 nm, λ_em_ 385 nm) AGE fluorescence measurement.

	Anti-vesperlysines-like AGE assay (mean ± S.D.)	Anti-pentosidine-like AGE assay (mean ± S.D.)
Z'-factor	0.70 ± 0.08	0.75 ± 0.07
SSMD	9.9 ± 1.5	11.1 ± 2.2
S/N	9.7 ± 1.8	16.1 ± 2.5
S/B	12.1 ± 4.1	13.4 ± 4.2
Separation band	4.4 ± 1.2 × 10^3^	4.5 ± 1.0 × 10^3^
Plate-to-plate variability (%)	7	5
Day-to-day variability (%)	3	3

**Figure 3:**
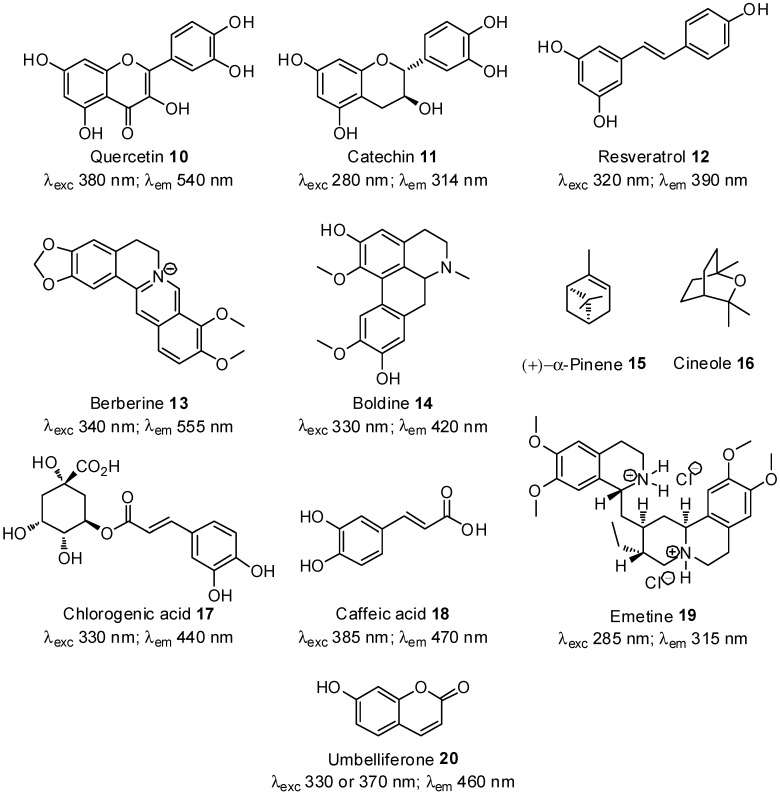
Tested NPs **10**–**20** and their fluorescent excitation and emission wavelengths in water.

In the present work, the anti-AGE activity of a small library of NPs was first evaluated with measurement of both vesperlysines-like and pentosidine-like AGEs to check the efficiency of this approach in overcoming fluorescence interference phenomena between AGEs and tested products. NPs previously described as being powerful AGE inhibitors (quercetin (**10**), catechin (**11**), resveratrol (**12**), berberine (**13**) and boldine (**14**)), inactive compounds (α-pinene (**15**) and cineole (**16**)), or which had given unexpected results (caffeic and chlorogenic acids **17** and **18**, emetine (**19**) and umbelliferone (**20**)), were chosen (Figure 3) [37]. The products were screened at 1 mg/mL and 0.1 mg/mL without replicates. Since such screening is usually aimed at selecting the most active substances, a suitable number of hits or minimal activity are predefined. In this case, we thus considered that most active products should be able to inhibit vesperlysines- or pentosidine-like AGE formation by at least 50%. After incubation with the tested compounds or extracts ("sample"), the remaining percentage of fluorescing AGEs was calculated as follows:

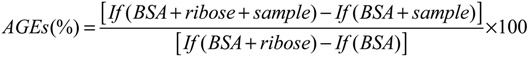
(1)


**Figure 4:**
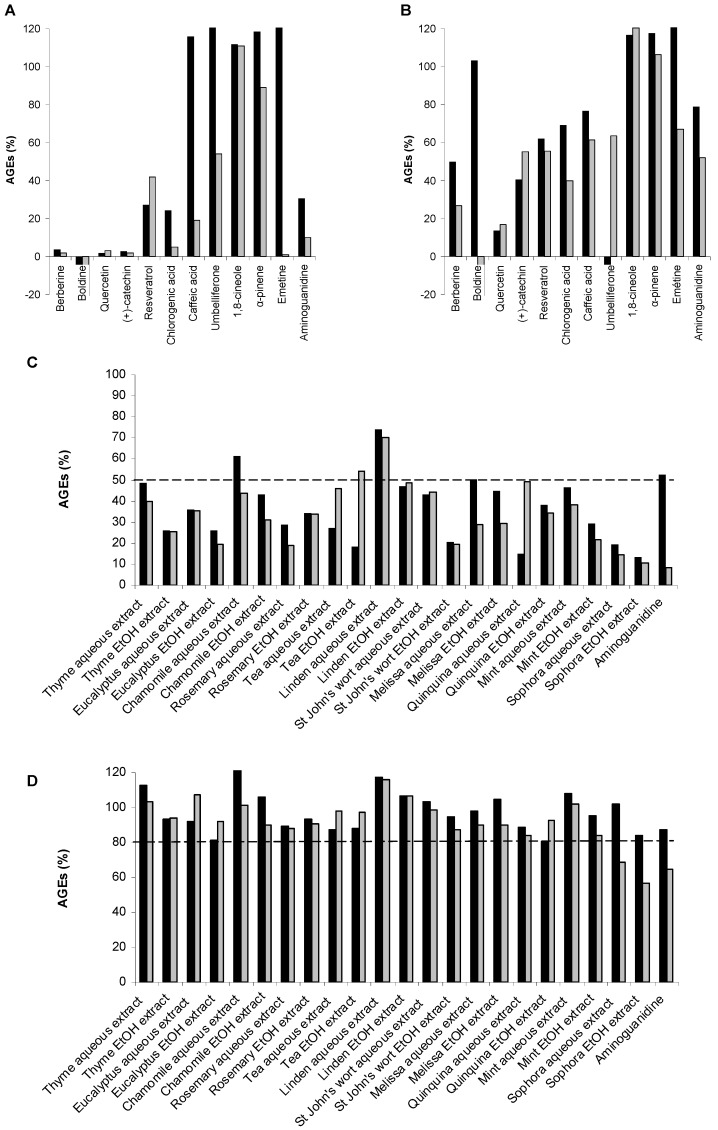
Vesperlysines-like (black) and pentosidine-like (grey) AGE formation in the presence of NPs (**A**: 1 mg/mL; **B**: 0.1 mg/mL) or plant extracts (**C**: 1 mg/mL; **D**: 0.1 mg/mL).

**Figure 5:**
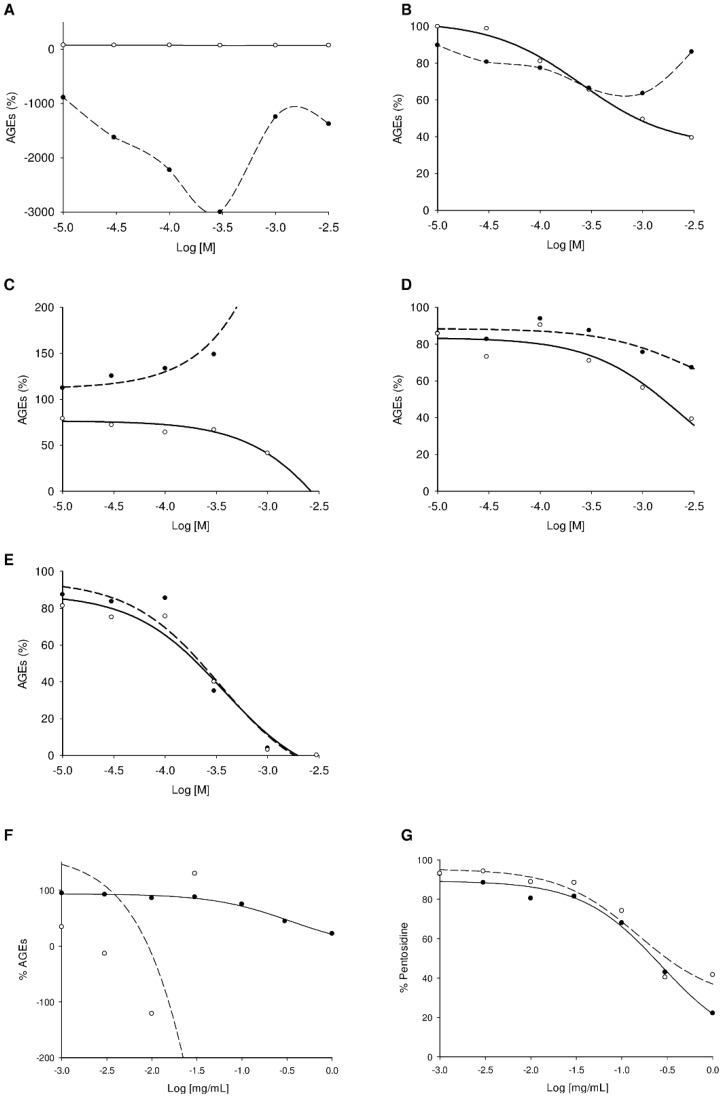
Dose-effect curves for vesperlysines-like AGE (dotted) and pentosidine-like AGE (bold) formation in the presence of umbelliferone **20** (**A**), caffeic acid **18** (**B**), emetine **19** (**C**) or aminoguanidine (**D**) or quercetin **10** (**E**) as well as dose-effect curves for vesperlysines-like AGE (**F**) and pentosidine-like AGE (**G**) formation in the presence of *Mammea neurophylla* fruit methanolic extract with (dotted) or without (bold) umbelliferone **20** (10% m/m).

As shown in Figure 4A/4B and Figure 5, after calculation, the AGE percentages were not always between 0 and 100%. Negative percentages were likely due to fluorescence collapse, *i.e.*, fluorescence of the sample alone was superior to that of AGEs and the sample. Very high values (>200%) could also be explained by fluorescence interference (see following explanation based on dose-effect curves for emetine **19**). AGE percentages of around 100% were associated with inactive compounds (*i.e.*, terpenes). As this assay was developed for a HTS with a Z'-factor of over 0.5, no replicates were needed, and therefore the observed percentages were sometimes slightly over 100.

When samples were tested at 1 mg/mL, most compounds were active except for well-known inactive terpenes. Evaluation at 0.1 mg/mL then appeared to be more selective since only four hits (quercetin (**10**), catechin (**11**) berberine (**13**), chlorogenic acid (**17**) and aminoguanidine, which is generally used as a reference compound in such assays, were identified. Correlatively, testing weakly concentrated samples (only 10 µg is required in this automated assay) could therefore enable evaluation of the anti-AGE potential of even trace amounts of NPs. This could be essential for the development of this screening process, since NPs are often isolated from plant extracts in small amounts.

Higher percentages of remaining AGEs were expected when the compounds were tested at lower concentration (Figure 4B). Actually this was not always the case [e.g., the caffeic acid (**18**) effect on vesperlysines-like AGE fluorescence]. Some explanations are given below and illustrated by U-shape dose-effect curves (Figure 5B, dotted line).

Overall, when NPs were tested, evaluation via fluorescence of vesperlysines- or pentosidine-like AGEs did not always give similar results. However, it is generally recognised that AGEs derived from the glycoxidation pathway (*i.e.*, CML, pentosidine or vesperlysines) are similarly affected independently of their respective structures [31,49]. This is why we believe that these differences reflect fluorescence interference between NPs and AGEs. In this regard, the following points should be noted.

First, fluorescent NPs could interfere with AGE fluorescence, as already mentioned. The fluorescence properties of NPs tested in water at pH 7.4 were also recorded and are given in Figure 3 [50,51,52,53,54,55,56,57,58]. As shown in Figure 4A, umbelliferone (**20**) tested at 1 mg/mL seemed inactive in the vesperlysines-like AGEs but inhibited pentosidine-like AGE formation by about 45%. When assessed at a 10-fold lower concentration, a negative value was obtained when vesperlysines-like AGEs were quantified, but pentosidine-like AGEs were inhibited by about 60%. The intense blue fluorescence of umbelliferone (λ_exc_ 330 or 370 nm; λ_em_ 460 nm) at neutral and basic pH [58,59] thus seemed to interfere with that of vesperlysines-like AGEs (λ_exc_ 370 nm; λ_em_ 440 nm). The dose-effect curve illustrating the vesperlysines-like AGE percentage as a function of the umbelliferone (**20**) concentration appeared as a U-shaped curve with negative values (Figure 5A, dotted line). On the contrary, the dose-effect curve illustrating the pentosidine-like AGE percentage (Figure 5A, bold line and Table 2) did not show any interference and indicated an IC_50_ > 3 mM. Umbelliferone here did not seem to influence AGE formation, as predicted by the initial screening (Figure 4A/B). The same phenomenon occurred when the anti-AGE effect of boldine (**14**) (λ_exc_ 330 nm; λ_em_ 420 nm) [54] was screened but fluorescence interference seemed to occur mostly with pentosidine-like AGEs (λ_exc_ 335 nm; λ_em_ 385 nm), as illustrated in Figure 4B. Regarding the fluorescence spectra, interference with vesperlysines- or pentosidine-like AGEs sometimes also occurs with resveratrol (**12**) [52], berberine (**13**) [53], chlorogenic acid (**17**) [55] and caffeic acid (**18**) [56]. In Figure 4B, for those compounds, the percentages of vesperlysines- or pentodisine-like AGEs differed markedly.

**Scheme 1:**
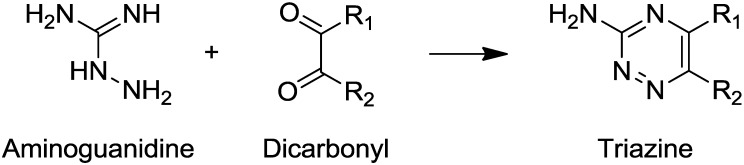
Formation of triazine derivatives from aminoguanidine and dicarbonyl compounds. This reaction explains how aminoguanidine can prevent AGEs formation by trapping dicarbonyl compounds.

Secondly, natural compounds and aminoguanidine could also react with ribose, BSA or their degradation products to form molecules able to interfere with vesperlysines- or pentodisine-like AGEs. This indicated that either those reaction products were fluorescent or their absorption spectrum was close to the excitation or emission wavelengths of AGEs. In Figure 4B, for emetine (**19**) and aminoguanidine, the percentages of vesperlysines- or pentodisine-like AGEs were very different. Aminoguanidine could react with dicarbonyls obtained by oxidation from ribose via Namiki or Wolff pathways [14] to form 1,2,4-triazine derivatives [60] (Scheme 1). 

**Table 2:** Effect of selected NPs and vegetal extracts on vesperlysines- and pentosidine-like AGE formation expressed as IC_50_ calculated from the corresponding sigmoidal dose-response curve [61].

Natural products	Effect on vesperlysines-like AGE formation (IC_50_, mM)	Effect on pentosidine-like AGEs formation (IC_50_, mM)
Quercetin **10**	0.2	0.2
(+)-catechin **11**	0.06	0.08
Resveratrol **12**	0.6	>3.0
Berberine **13**	0.4	0.2
Boldine **14**	0.5	^a^
Chlorogenic acid **17**	1.0	0.1
Caffeic acid **18**	>3.0	1.0
α-pinene **15**	>3.0	>3.0
1,8-cineole **16**	>3.0	>3.0
Emetine **19**	^a,b^	0.7
Umbelliferone **20**	^a^	>3.0
Aminoguanidine ^c^	10.0	2.0
**Plant extracts**	**Effect on vesperlysines-like AGE formation** **(IC_50_, mg/mL)**	**Effect on pentosidine-like AGE formation** **(IC_50_, mg/mL)**
Thyme aqueous extract	0.9	0.6
Thyme EtOH extract	0.3	0.4
Eucalyptus aqueous extract	0.4	0.5
Eucalyptus EtOH extract	0.3	0.3
Chamomille aqueous extract	>1.0	0.6
Chamomille EtOH extract	0.5	0.3
Rosemary aqueous extract	0.2	0.3
Rosemary EtOH extract	0.3	0.6
Tea aqueous extract	0.3	1.0
Tea EtOH extract	0.2	>1.0
Linden aqueous extract	>1.0	>1.0
Linden EtOH extract	>1.0	>1.0
St John’s wort aqueous extract	0.4	0.3
St John’s wort EtOH extract	0.3	0.2
Melissa aqueous extract	>1.0	0.3
Melissa EtOH extract	>1.0	0.8
Quinquina aqueous extract	0.2	0.2
Quinquina EtOH extract	0.1	0.1
Mint aqueous extract	0.7	0.5
Mint EtOH extract	0.3	0.2
Sophora aqueous extract	0.4	0.1
Sophora EtOH extract	0.2	0.1
Aminoguanidine ^c^	>1.0	0.2
Quercetin ^c^	0.06	0.06

^a^ No sigmoidal curve; ^b^ AGE inducer; ^c^ Reference compounds [37].

The UV spectrum of this heterocyclic moiety has several absorption bands between 350 nm and 430 nm [62] and, for aromatic derivatives, emission fluorescence can be observed around 460–480 nm (λ_exc_ 275 nm). The similar photophysical behaviour of such 1,2,4-triazines and vesperlysines-like AGEs could explain why vesperlysines-like AGEs seemed to be present in higher quantity than pentosidine-like AGEs on the dose-effect curves (Figure 5D). Such interference due to a reaction between BSA, ribose or its oxidation products and the tested compound could also be involved in the U-shape curves (vesperlysines-like AGEs) obtained with phenolic acids **17** and **18** (Figure 5B).

Finally, the authors previously described emetine (**19**) as being a potential inducer of AGE formation when vesperlysines-like AGEs were quantified [37]. Paradoxically, **19** here seemed to inhibit pentosidine-like AGEs (Figure 4 and Figure 5C). The UV (λ_max_ 236 and 283 nm) and fluorescent (λ_exc_ 285 nm; λ_em_ 315 nm) spectra of **19** [57] did not suggest any direct interference with AGEs fluorescing at 440 nm (λ_exc_ 370 nm). However, emetine (**19**), a tetrahydroisoquinoline, could easily react with carbonyl residues present in the mixture to form a reactive enamine [63] suitable to form fluorescent heterocycles interfering with AGE fluorescence.

Moreover, although soluble in DMSO, natural products tested in aqueous solution could precipitate and modify light diffusion or fluorescence emission.

The IC_50_ value was calculated when a sigmoid dose-effect curve was obtained (Table 2). The obtained values confirmed the preliminary screening results since quercetin (**10**), catechin (**11**), berberine (**13**) and chlorogenic acid (**17**) appeared to be the best anti-AGE compounds.

Concerning the anti-AGE assays based on fluorescence at 440 nm (λ_exc_ 370 nm), aminoguanidine was usually chosen as the reference compound because of its clinical development. However, considering that the quercetin (**10**) IC_50_ was lower than the aminoguanidine IC_50_ and that aminoguanidine tends to form heterocycles interfering with AGE fluorescence at 440 nm, quercetin (**10**) should likely be selected as a reference compound in such HTS assays.

### 2.4. Screening of the Antivesperlysines-Like and Antipentosidine-Like AGE Activity of Plant Extracts and Validation of Hits via Dose-Effect Curves

The aim of this study was to develop an improved version of the anti-AGE assay previously described by Derbré *et al.* [37] suitable for screening complex plant extracts. We then evaluated 11 commonly used food and medicinal plants selected for their capacity to produce various secondary metabolites (*i.e.*, terpenes, polyphenols and alkaloids). Their phytochemistry and uses have been well described [64]. Some of those plants were selected because their NPs (*i.e.*, polyphenols) have been described as being antioxidant or dicarbonyl scavengers, and could thus be potent anti-AGEs [14]. Aqueous and ethanolic extracts were obtained using pressurized liquid extraction.

**Figure 6:**
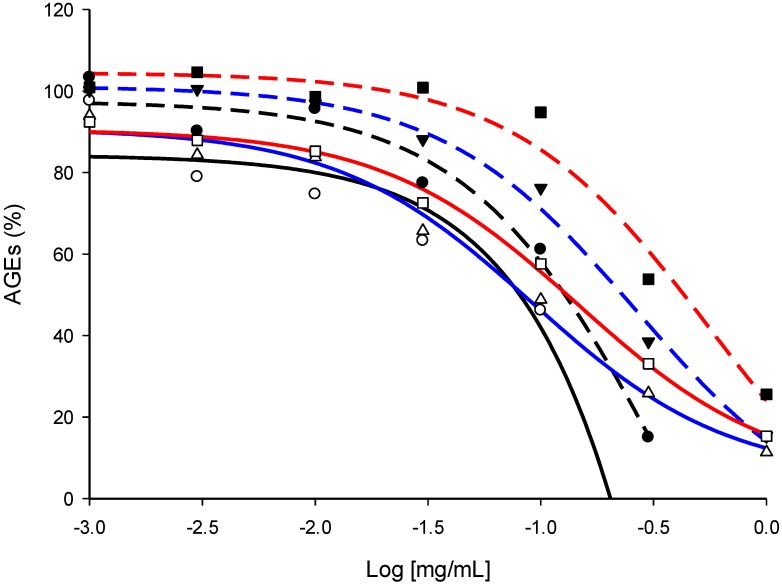
Dose-effect curves for vesperlysines-like AGE (dotted) and pentosidine-like AGE (bold) formation in the presence of quinquina EtOH extract (Black), sophora EtOH extract (Blue) or sophora aqueous extract (Red).

As for pure NPs, the antivesperlysines-like and antipentosidine-like AGEs activity of each extract was screened at 1 and 0.1 mg/mL. As illustrated in Figure 4C at 1 mg/mL each extract—except linden aqueous extract—inhibited vesperlysines-like and/or pentosidine-like AGE formation by at least 50%. At 0.1 mg/mL, none of them prevented AGE formation by more than 50%. Therefore, extracts preventing vesperlysines- or pentosidine-like AGE formation by more than 20% were considered as hits, *i.e.*, sophora (ethanolic and aqueous) and quinquina (ethanolic) emerged as the best anti-AGE extracts. This was confirmed by dose-effect curves and IC_50_ measurements (Figure 6). These three extracts showed the best IC_50_ (~0.1 mg/mL) when the effects on vesperlysines-like or pentosidine-like AGEs were assessed (Table 2). These results seemed particularly relevant since antioxidant rutin (quercetin-3-*O*-rutinoside) and catechin polymers are known to be major compounds of sophora buds and quinquina bark, respectively. These secondary metabolites were found to be potent anti-AGEs, as described above.

To demonstrate the advantages of both vesperlysines-like and pentosidine-like AGEs measurement, the fluorescent and inactive coumarin umbelliferone (**20**, 10% m/m) was added to a previously described anti-AGEs extract (*Mammea neurophylla*, Clusiaceae). The IC_50_ of this methanolic fruit extract was previously calculated to be 0.18 mg/mL [65] when vesperlysines-like AGEs were quantified. As expected, there was strong interference when umbelliferone was added (Figure 5F). However, when pentosidine-like AGEs were measured, the same curves were obtained when the same extract was used with or without umbelliferone (Figure 5G).

Moreover, as expected and depicted in Figure 4C/D, the plant extracts interfered with vesperlysines-like and/or pentosidine-like AGEs since their percentages, as evaluated using fluorescence, often differed markedly. As an example, for tea extracts (0.1 and 1 mg/mL), higher values were obtained when fluorescence was measured at 385 nm (λ_exc_ 335 nm). This could be directly linked with the high amounts of flavan-3-ols mono- and oligomers (*i.e.*, procyanidins) previously detected in tea extracts [66,67]. Indeed, the same difference was obtained for catechin, a flavan-3-ol (Figure 4B). This illustrates the relevance of this assay and also the importance of carefully checking the fluorescence of different AGEs in such a HTS.

## 3. Experimental

### 3.1. Chemicals and Materials

Bovine serum albumin (BSA, fraction V), potassium phosphate monobasic, potassium phosphate dibasic trihydrate, sodium azide, aminoguanidine hydrochloride were purchased from Sigma-Aldrich (St Quentin Fallavier, France). Ribose was from Alfa Aesar (Schiltigheim, France). Commercial natural products were purchased from Sigma-Aldrich or Extrasynthèse (Genay, France).

Fluorescence spectroscopy measurements, steady-state excitation and emission spectra of vesperlysines- and pentosidine-like AGEs formed from BSA (10 mg/mL) and ribose (0.5 M) were recorded on a Quantamaster spectrometer (PTI, Birmingham, NJ, USA) set at a 2 nm band pass for both the excitation and emission collection monochromator. The emitted light was detected at a 90° angle with respect to the excitation beam.

Ninety-six well black bottom plates and their silicone lids were from Greiner Bio One (Fisher Scientific, Illkirch, France). The automated 96-well microtiter plate assay was conducted on a Freedom Evo® 100 liquid handling workstation (Tecan, Lyon, France). The liquid handling (LiHa) arm was equipped with four LiHA standard fixed washable tips (Teflon^®^-coated stainless steel, resistant to DMSO, Tecan). Dispensing steps, *i.e.*, liquid class parameters, were optimized and programmed using Evoware software. AGE fluorescence was measured using a microplate spectrofluorometer infinite M200 (Tecan, Lyon, France) and Magellan software (Tecan).

### 3.2. Plant Extractions Using Pressurized Solvents

Dried herbs (leaves from *Camellia sinensis* (L.) Kuntze, *Eucalyptus globulus* Labill., *Melissa officinalis* L., *Mentha piperita* L. and *Rosmarinus officinalis* L.; flowers from *Chamaemelum nobile* (L.) All. and *Tilia cordata* Mill.; flowering tops from *Hypericum perforatum* L. and *Thymus vulgaris* L.; flower buds from *Styphnolobium japonicum* L. and bark from *Cinchona pubescens* Vahl.) were obtained from Herboristerie Cailleau (Chemillé, France) or Promoplantes (Chanzeaux, France). Each sample was pulverized and 10 g were mixed with an equivalent amount (m/m) of diatomaceous earth before extraction by pressurized liquid extraction (PLE) with a SpeedExtractor E-914 (Büchi, Rungis, France). The material was extracted in 40 mL PLE cells with either ethanol or deionised water using the following parameters: temperature 100 °C, pressure 100 bar, 3 cycles (1st cycle: heat up 5 min, discharge 2 min; 2nd and 3rd cycles: heat up 1 min, hold 10 min, discharge 2 min), and flush with gas (N_2_, 2 min). Crude extracts were concentrated at 40 to 60 °C under reduced pressure to obtain a dry extract.

### 3.3. Automated Anti-AGE Screening

Automated screening of natural products (NPs) or plant extracts was performed. Pure compounds or extracts were prepared in DMSO stock solutions at 1 mg/mL. 80 products or extracts were stored in 96-well round bottom plates (3363) covered with Corning costar® lids (Fisher Scientific).

The assay involved incubating BSA (10 mg/mL) with d-ribose (0.5 M) and the tested compound or extract (0.1 mg/mL) in phosphate buffer, 50 mM, pH 7.4 (NaN_3_ 0.02%). Solutions were incubated in black 96-well microtiter plates at 37 °C for 24 h in a closed system before AGE fluorescence measurement (40 extracts or products tested per plate). To subtract the tested product or extract intrinsic fluorescence, the fluorescence resulting from incubation, under the same conditions of BSA (10 mg/mL) and the tested compound or extract (0.1 mg/mL) was subtracted for each measurement. A control, *i.e.*, 100% AGE formation, consisted of wells with BSA (10 mg/mL) and d-ribose (0.5 M). A blank control, with no AGE formation, consisted of wells with only BSA. The final assay volume was 100 μL. Both vesperlysines-like (λ_exc_ 370 nm; λ_em_ 440 nm) and pentosidine-like (λ_exc_ 335 nm; λ_em_ 385 nm) AGE fluorescence were measured using a microplate spectrofluorometer. For each compound or extract (0.1 mg/mL), the percentage of AGE formation was calculated as follows: AGEs (%) = [fluorescence intensity (sample) − fluorescence intensity (blank of sample)]/[fluorescence intensity (control) − fluorescence intensity (blank of control)] × 100. The best compounds or extracts were selected for further determination of the concentration inhibiting 50% AGE formation (IC_50_).

### 3.4. Determination of Extract or Product Concentration Inhibiting 50% AGE Formation (IC_50_) Using Liquid Handling Facilities

The IC_50_ was determined using a previously described method [68] with slight modifications. The assay involved incubating BSA (10 mg/mL) with d-ribose (0.5 M) and the tested compound (3 µM to 3 mM) or extract (1 µg to 1 mg) in 50 mM phosphate buffer at pH 7.4 (NaN_3_ 0.02%). Solutions were incubated in 96-well microtiter plates at 37 °C for 24 h in a closed system before AGE fluorescence measurement. Fluorescence resulting from the incubation, under the same BSA (10 mg/mL) and tested compound (3 µM to 3 mM) or extract (1 µg to 1 mg) conditions, was subtracted for each measurement. A control, *i.e.*, no inhibition of AGE formation, consisted of wells with BSA (10 mg/mL) and d-ribose (0.5 M). A blank of control, *i.e.*, 100% inhibition of AGE formation, consisted of wells with only BSA. The final assay volume was 100 μL. Both vesperlysines-like (λ_exc_ 370 nm; λ_em_ 440 nm) and pentosidine-like (λ_exc_ 335 nm; λ_em_ 385 nm) AGE fluorescence were measured using a microplate spectrofluorometer. The percentage of AGE formation was calculated as follows for each compound/extract concentration: AGEs (%) = [fluorescence intensity (sample) − fluorescence intensity (blank of sample)]/[fluorescence intensity (control) − fluorescence intensity (blank of control)] × 100. Dose-effect curves were best fit with a sigmoidal dose-response equation using Sigma Plot 12.0 software, which enabled calculation of the IC_50_ values.

### 3.5. Assay Quality Determination Using the Z'-Factor

All statistical analyses were performed as previously reported [37]. In particular, the Z'-factor was calculated as follows [46]:

Z' = 1 − (3 × σ_c+_ + 3 × σ_c−_)/|µ_c+_ − µ_c−_| (2)

where σ_c+_, µ_c+_, σ_c−_, µ_c−_ represent the standard deviations (σ) and means (µ) of the maximum (c+) and minimum (c−) signals.

Maximum or minimum signals were obtained by incubating BSA (10 mg/mL) with d-ribose (0.5 M) or BSA (10 mg/mL), respectively, in 50 mM phosphate buffer at pH 7.4 (NaN_3_ 0.02%). Solutions (100 μL) were incubated in black 96-well microtiter plates at 37 °C for 24 h in a closed system before vesperlysines-like (λ_exc_ 370 nm; λ_em_ 440 nm) or pentosidine-like (λ_exc_ 335 nm; λ_em_ 385 nm) AGE fluorescence measurement using a microplate spectrofluorometer.

### 3.6. Assay Quality Determination Using SSMD

Signal-to-noise ratio, signal-to-background ratio, and Z-factor have been adopted as quality control (QC) metrics in HTS assays. More recently, Zhang proposed strictly standardized mean difference (SSMD, denoted as *β*) as a QC metrics in RNAi HTS assays. SSMD is defined as the ratio of mean to standard deviation of the difference between 2 populations. It was calculated as follows [47,48]:

*β* = |µ_c+_ − µ_c−_|/(σ_c+_² + σ_c−_²)^1/2 ^ (3)

## 4. Conclusions

We previously reported an anti-AGE HTS assay based on vesperlysines-like AGE fluorescence obtained after 24 h incubation of BSA and ribose in physiological conditions. An improved assay was developed here in order to overcome previously described fluorescence interference. This involved the simultaneous quantification of both vesperlysines-like (λ_exc_ 370 nm; λ_em_ 440 nm) and pentosidine-like (λ_exc_ 335 nm; λ_em_ 385 nm) AGEs. In a batch of medicinal and food plant extracts, hits were selected when the fluorescence decreased for at least one wavelength. As in many HTS assays, the hits should be further confirmed by biological methods. As a confirmation of the viability of our assay, we noted that the active extracts revealed during the assay also contained powerful anti-AGE substances. We also demonstrated that this assay could be applied to a plant extracts screened at 0.1 mg/mL and, consequently, to bioguided semi-preparative HPLC fractionation. This assay could therefore be useful for exploring the high chemodiversity of NPs regarding their anti-AGE potential. This could lead to the development of plant extracts to serve as dietary supplements or to enhance diabetes diets. Finally, quercetin (**10**) was found to be a more appropriate reference than aminoguanidine when vesperlysines-like AGEs were quantified using fluorescence.
